# Vector competence of *Anopheles quadrimaculatus* and *Aedes albopictus* for genetically distinct Jamestown Canyon virus strains circulating in the Northeast United States

**DOI:** 10.1186/s13071-022-05342-3

**Published:** 2022-06-23

**Authors:** Constentin Dieme, Laura D. Kramer, Alexander T. Ciota

**Affiliations:** 1 Institut Pasteur de Guinée, Conakry, Guinea; 2 Wadsworth Center, New York State Department of Health, Slingerlands, NY USA; 3 Department of Biomedical Sciences, School of Public Health, State University of New York at Albany, Albany, NY USA

**Keywords:** Jamestown Canyon virus, *Aedes albopictus*, *Anopheles quadrimaculatus*, Vector competence

## Abstract

**Background:**

Jamestown Canyon virus (JCV; Peribunyaviridae, Orthobunyavirus) is a mosquito-borne pathogen belonging to the California serogroup. The virus is endemic in North America and increasingly recognized as a public health concern. In this study, we determined the vector competence of *Anopheles* (An.) *quadrimaculatus* and *Aedes* (Ae.) *albopictus* for five JCV strains belonging to the two lineages circulating in the Northeast.

**Methods:**

*An. quadrimaculatus* and *Ae. albopictus* were fed blood meals containing two lineage A strains and three lineage B strains. Vector competence of both mosquito species was evaluated at 7- and 14-days post-feeding (dpf) by testing for virus presence in bodies, legs, and saliva.

**Results:**

Our results demonstrated that *Ae. albopictus* mosquitoes are a competent vector for both lineages, with similar transmission levels for all strains tested. Variable levels of infection (46–83%) and dissemination (17–38%) were measured in *An. quadrimaculatus*, yet no transmission was detected for the five JCV strains evaluated.

**Conclusions:**

Our results demonstrate that establishment of *Ae. albopictus* in the Northeast could increase the risk of JCV but suggest *An. quadrimaculatus* are not a competent vector for JCV.

**Graphical Abstract:**

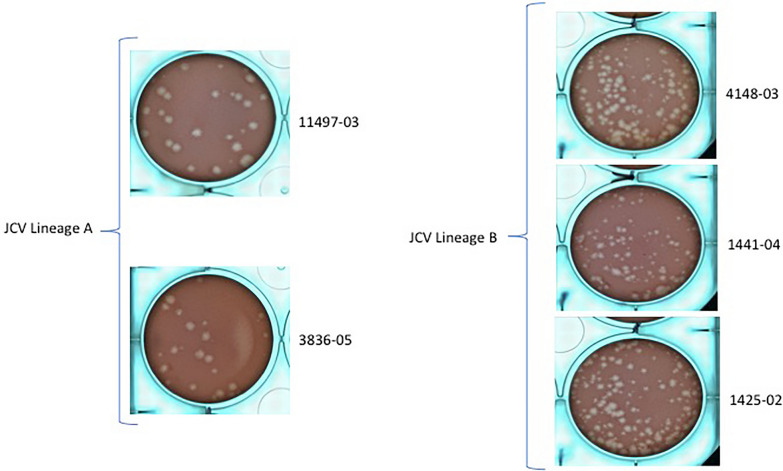

## Background

Jamestown Canyon virus (JCV; Peribunyaviridae, Orthobunyavirus) belongs to the California serogroup with La Crosse virus (LACV) and snowshoe hare virus (SSHV) [1]. JCV is broadly distributed throughout the USA and Canada [2]. The genome of JCV is comprised of three single-stranded segments of negative-sense RNA designated as the small (S), medium (M), and large (L), encoding the nucleocapsid, surface glycoprotein precursor, and RNA polymerase, respectively [3–5]. JCV isolates fall into two lineages, designated A and B [3, 4]. Both lineages have been detected in Connecticut and Massachusetts [2, 4, 5]; however, only circulation of lineage A has been reported in New York State (NYS) [6].

The number of JCV cases in the US has increased in recent years [2, 7]. The virus affects mostly adults and can cause acute febrile illness, severe meningitis, and encephalitis [2, 8, 9]. Despite the high incidence of neuroinvasive disease cases (54–79%), mortality is rare, with only five total deaths reported in the US [7, 10, 11]. In NYS, JCV has been documented since 1971 with at least four confirmed human cases [11, 12]. Moreover, sera from patients with and without central nervous system (CNS) disease revealed that JCV is the most prevalent arbovirus in NYS patients with antibody to California serogroup viruses, followed by LACV [13]. A previous study in Connecticut demonstrated seroprevalence rates for JCV ranging from 3.9–10.1%, yet recent increases in diagnosed cases would suggest seroprevalence likely exceeds historic rates [14, 15].

JCV infects a variety of free-ranging ungulates (deer, bison, moose, elk), but white-tailed deer are recognized as a principal amplification host [3, 8]. The seroprevalence rates of white-tailed deer for JCV were estimated to be 21% and 54.7% in Connecticut and NYS, respectively [16, 17]. In NYS, the virus was found associated with 12 mosquito species including *An. punctipennis* and *An. quadrimaculatus*, which regularly feed on white-tailed deer [6, 18]. In total, JCV has been isolated from 26 mosquito species in several genera including *Aedes* (Ae.), *Anopheles* (An.), *Culex*, *Coquillettidia*, and *Culiseta* [17, 19]. While it has been suggested that *Aedes* mosquitoes act as principal vectors [17], the primary vector of JCV in the Northeast, including in NYS, is unknown. Isolation of JCV from *Ae. albopictus* and *An. quadrimaculatus* is suggestive of their potential involvement in the transmission cycle of the virus [17]. Furthermore, *Anopheles* spp. have been shown to play a primary role in vectoring the orthobunyavirus Cache Valley virus (CVV) in NYS [20, 21].

The objective of this study was to determine the vector competence of *An. quadrimaculatus* and *Ae. albopictus* for JCV strains belonging to the two lineages circulating in the Northeast.

## Methods

### Mosquitoes

A colony of unknown generations of *An. quadrimaculatus* (Orlando strain) was obtained from BEI (MRA-139) and were maintained at 27 °C under standard rearing [22, 23]. *Aedes albopictus* colony (Spring Valley, New York, kindly provided by Laura Harrington, Cornell University) was established in 2019 from field-collected eggs. *Aedes albopictus* were hatched in distilled water, reared and maintained similarly to *Anopheles* as described [23].

### Experimental infections of mosquitoes with Jamestown Canyon virus

Five JCV strains originally isolated from mosquito pools in Connecticut (kindly provided by Philip Armstrong, CAES), and previously found to be genetically distinct, were freshly propagated in Vero (African Green Monkey kidney) cell cultures in separated plates maintained at 37 °C, 5% CO_2_. These included 3836–05 (lineage A, 2005; S, M, L accession nos. EF681835, EF687050, EF687103), 11,497–03 (lineage A, 2003; S, M, L accession nos. EF81845, EF87121, EF687059), 1441–01 (lineage B, 2001; S, M, L accession nos. EF681814, EF687110, EF687027), 1425–02 (lineage B, 2002; S, M, L accession nos. EF681813, EF687091, EF687039) and 4148–03 (lineage B, 2003; S, M, L accession nos. EF681827, EF687114, EF687051). At 48 h following infection (multiplicity of infection ≈ 1.0), the supernatant of each strain was harvested and diluted 1:1 with defibrinated sheep blood plus a final concentration of 2.5% sucrose. Female *An. quadrimaculatus* mosquitoes (3–5 days old) from unknown generations were deprived of sugar for 1–2 h, and F7 female *Ae. albopictus* (5–7 days old) deprived of sugar for 24 h were offered a JCV-blood suspension for 45 min via a Hemotek membrane feeding system (Discovery Workshops, Acrington, UK) with porcine sausage casing membrane at 37 °C [22]. Following feeding, females were anesthetized with CO_2_, and fully engorged mosquitoes were transferred to 0.6-l cardboard containers and maintained with 10% sucrose at 27 °C until harvested for testing [24]. A 1-ml aliquot of each pre-feeding blood meal was frozen at − 80 °C to determine JCV blood meal titers.

### Evaluation of mosquito vector competence for Jamestown Canyon virus

Infection, dissemination, and transmission were determined on days 7 and 14 post-infectious blood (dpi) meal as previously described [22, 23]. Blood meals, mosquito bodies, legs and salivary secretions were assayed for infection by plaque assay on Vero cells [23, 24]. Briefly, Vero cells were seeded in six-well plates at a density of 6.0 × 10^5^ cells per well and were incubated for 3–4 days at 37 °C in 5% CO_2,_ to produce a confluent monolayer. The cell monolayers were inoculated with 0.1-ml of tenfold serial dilutions of the blood meals (diluted in BA-1) in duplicate or with undiluted mosquito bodies, legs and salivary secretions from each homogenized mosquito sample. Viral adsorption was allowed to proceed for 1 h at 37 °C with rocking of the plates every 15 min. A 3-ml overlay of MEM, 5% FBS and 0.6% Oxoid agar supplemented with 0.2 × penicillin/streptomycin per ml, 0.5 μg fungizone (Amphotericin B) per ml and 20 ug gentamicin per ml was added at the conclusion of adsorption. The infected monolayers were incubated at 37 °C in 5% CO_2_. After 2 days of infection, a second overlay, similar to the first but with the addition of 1.5% neutral red (Sigma-Aldrich Co., St. Louis, MO), was added to the wells, and the plates were incubated at 37 °C in 5% CO_2_ overnight. For the blood meal, the plaques were counted, and the viral titer was calculated and expressed as plaque-forming units (PFU) per ml. For mosquito samples, presence or absence of plaques was checked.

Dissemination rate is the proportion of mosquitoes with infected legs among the mosquitoes with infected bodies. Transmission rate is the proportion of mosquitoes with infectious saliva collected by capillary transmission method among mosquitoes with disseminated infection [22, 23].

### Statistical analysis

A Fisher’s exact test was used to compare infection, dissemination and transmission rates within mosquito species and between both time points and virus strains [21, 23]. All statistical analyses were carried out at a significance level of *p* < 0.05. OpenEpi, version 3, open-source calculator-TwobyTwo (http://www.openepi.com/TwobyTwo/TwobyTwo.htm) was used for all statistical analysis.

## Results

*Anopheles quadrimaculatus* mosquitoes were orally exposed to five genetically distinct JCV strains (two lineage A [3836–05, 11,497–03] and three lineage B [1441–04, 1425–02, 4148–03]). Pairwise percent identity for these strains ranges from 99.6 to 88.6 across segments, with distinct mutations identified in each segment for all strains. Viral load of blood meals ranged from 6.2 to 6.6 log_10_ PFU/ml. Mosquitoes were processed at days 7 and 14 dpi to determine infection, dissemination and transmission rates. Infection rates of *An. quadramaculatis* at 7 dpi ranged from 70.00 to 83.33% and were statistically similar among strains. Interestingly, an overall trend of decreasing infection was measured between time points for all strains, with the exception of 4148–03. This decrease of infection rate was only significant for the 1425–02 strain (83.33% vs. 46.67%, Fisher’s exact test, *P* < 0.003, OR: 5.714, 95% CI 1.526, 23.7; Table 1). In addition, a significant difference in infection rates was measured at 14 dpi between strains 1425–02 and 4148–03 (46.67% vs. 76.67%, respectively; Fisher’s exact test, *P* = 0.016, OR: 3.755, 95% CI 1.097–13.43; Table 1). Except for the 1441–04 strain, which was not disseminated at 7 dpi, similar dissemination rates were observed within strains and between time points. No transmission was detected for any JCV strain at 7 or 14 dpi for *An. quadramaculatis* (Table 1).

**Table 1:** Vector competence of *Anopheles quadrimaculatus* for genetically distinct Jamestown Canyon virus strains

	Lineage	Strain	Input_a_	7 dpi			14dpi		
				IRs	DRs	TRs	IRs	DRs	TRs
	Lineage A	3836–05	6.5	76.67 (23/30)	26.09 (6/23)	0.00 (0/6)	56.67 (17/30)	35.29 (6/17)	0.00 (0/6)
	Lineage A	11,497–03	6.6	70.00 (21/30)	23.81 (5/21)	0.00 (0/5)	53.33 (16/30)	25.00 (4/16)	0.00 (0/4)
	Mean ± SD	NA	NA	73.33 ± 4.72	24,95 ± 1.61	0.00	55.00 ± 2.36	30.14 ± 7.28	0.00
*Anopheles quadrimaculatus*	Lineage B	1441–04	6.2	70.00 (21/30)	0.00 (0/21)^b^	0.00 (0/0)	66.67 (20/30)	30.00 (6/20)	0.00 (0/6)
	Lineage B	1425–02	6.4	83.33 (25/30)	24.00 (6/25)	0.00 (0/6)	46.67 (14/30)^b^	21.43 (3/14)	0.00 (0/3)
	Lineage B	4148–03	6.5	70.00 (21/30)	38.10 (8/21)	0.00 (0/8)	76.67 (23/30)^c^	17.39 (4/23)	0.00 (0/4)
	Mean ± SD	NA	NA	74.44 ± 7.70	20.70 ± 19.26	0.00	63.34 ± 15.27	22.94 ± 6.44	0.00

*dpi* days post-infection, *IR* infection rate, *DR* dissemination rate, *TR* transmission rate

^a^blood meal titer (log_10_ pfu/ml)

^b^Significant difference in the infection rates between time points for the 1425–02 and 1441–04 strains

^c^Significant difference in the infection rates between 1425–02 and 4148–03 strains at 14 dpi

Following *Ae. albopictus* exposure to similar JCV blood meal titers (6.5–7.1 log_10_ PFU/ml), high rates of infection (90.00–100% at 7 dpi, 96.67–100% at 14 dpi) and dissemination (96.30–100% at 7dpi, 93.10–100% at 14 dpi) were measured, with no statistical differences between strains and time points (Table 2). Overall, a significant effect of species was measured independent of strain, with higher infection, dissemination and transmission rates at both time points in *Ae. albopictus* relative to *An. quadramaculatis* (two-way ANOVA, *P* < 0.05). All JCV strains were transmitted at 7 dpi by *Ae. albopictus*. A decrease in transmission rates with time was measured for all strains, with no detectable virus in saliva of two strains (3836–05 and 1441–04) at 14 dpi. Significantly lower infection rates were measured following exposure to blood meals with lower input titers (4.7 to 5.2 log_10_ PFU/ml; Fisher’s exact test, *P* < 0.0001, OR: 10.8, 95% CI 2.168–68.39). Interestingly, following both high and low dose exposure, infected *Ae. albopictus* showed high dissemination rates (96.30–100% vs. 85.71–100% at 7 dpi and 100% at 14 dpi for both doses). Similar transmission rates were also measured at 7 and 14 dpi for both lineages, with one strain of each lineage not transmitted at 14 dpi. Our results indicate that *Ae. albopictus* mosquitoes are a highly competent vector for both JCV lineages (Table 2).

**Table 2:** Vector competence of *Aedes albopictus* for genetically distinct Jamestown Canyon virus strains

	Lineage	Strain	Input^a^	7 dpi			14dpi		
				IRs	DRs	TRs	IRs	DRs	TRs
	Lineage A	3836–05	7.1	96.67 (29/30)	100.00 (29/29)	13.79 (4/29)	96.67 (29/30)	93.10 (27/29)	0.00 (0/27)
	Lineage A	11,497–03	7.0	93.33 (28/30)	100.00 (28/28)	14.29 (4/28)	100.00 (30/30)	100.00 (30/30)	3.33 (1/30)
	Mean ± SD	NA	NA	95.00 ± 2.36	100.00	14.04 ± 0.35	98.33 ± 2.35	96.55 ± 4.88	1.66 ± 2.35
	Lineage B	1441–04	6.5	90.00 (27/30)	96.30 (26/27)	15.38 (4/26)	100.00 (30/30)	100.00 (30/30)	0.00 (0/30)
	Lineage B	1425–02	6.6	100.00 (30/30)	100.00 (30/30)	30.00 (9/30)	100.00 (30/30)	100.00 (30/30)	3.33 (1/30)
	Lineage B	4148–03	6.6	100.00 (30/30)	100.00 (30/30)	30.00 (9/30)	100.00 (25/25)	100.00 (25/25)	12.00 (3/25)
*Aedes albopictus*	Mean ± SD	NA	NA	96.67 ± 5.77	98.77 ± 2.14	25.13 ± 8.44	100.00	100.00	5.11 ± 6.19
	Lineage A	3836–05	4.9	42.86 (9/21)^b^	100.00 (9/9)	27.27 (3/11)	37.5 (9/24)^b^	100.00 (9/9)	0.00 (0/9)
	Lineage A	11,497–03	5.2	20.83 (5/24)^b^	100.00 (5/5)	20.00 (1/5)	18.51 (5/27)^b^	100.00 (5/5)	20.00 (1/5)
	Mean ± SD	NA	NA	31.84 ± 15.58	100.00	23.63 ± 5.14	28.00 ± 13.42	100.00	10.00 ± 14.14
	Lineage B	1441–04	4.7	45.45 (10/22)^b^	100.00 (10/10)	20.00 (2/10)	45.45 (10/22)^b^	100.00 (10/10)	0.00 (0/10)
	Lineage B	1425–02	4.8	33.33 (7/21)^b^	85.71 (6/7)	28.57 (2/7)	36.00 (9/25)^b^	100.00 (9/9)	11.11 (1/9)
	Lineage B	4148–03	5.1	45.45 (10/22)^b^	100.00 (10/10)	20.00(2/10)	42.11 (8/19)^b^	100.00 (8/8)	12.50 (1/8)
	Mean ± SD	NA	NA	41.41 ± 7.00	95.24 ± 8.25	22.86 ± 4.95	41.19 ± 4.79	100.00	7.87 ± 6.85

*dpi* days post-infection, *IR* infection rate, *DR* dissemination rate, *TR* transmission rates

^a^blood meal titer (log_10_ pfu/ml)

^b^Significant difference in the infection rates following exposure to blood meals with lower input titers within strain by time point

## Discussion

With the increase of JCV cases in the US, it is important to identify mosquito species that are implicated in virus transmission to better understand the epidemiological risk in the Northeast and adapt vector control strategies. Thus, we assessed the vector competence of the most abundant *Anopheles* species (*An. quadrimaculatus*) and the most abundant invasive species (*Ae. albopictus*) in NYS for both JCV lineages circulating in the Northeast [20, 21]. Our results suggest that *Ae. albopictus* is highly competent for JCV but *An. quadrimaculatus* is not.

Recent studies demonstrated that *An. quadrimaculatus* mosquitoes are able to transmit viruses such as Mayaro virus and CVV [20, 23, 25, 26]. In the US, JCV has been consistently isolated from at least four *Anopheles* species including *An. punctipennis*, *An. quadrimaculatus*, *An. crucians* and *An. walkeri* [17]. In the Northeast, JCV is regularly detected from *An. punctipennis* and *An. walkeri* in Connecticut [17, 19], from *An. punctipennis*, *An. quadrimaculatus* and *An. walkeri* in Massachusetts [27] and from *An. punctipennis* and *An. quadrimaculatus* in NYS [6]. Consistent isolation of JCV from *An. quadrimaculatus* could either suggest that this species is involved in virus transmission or simply be a reflection of frequent feeding on competent hosts such as white-tailed deer [18]. Heard et al. showed *An. punctipennis* mosquitoes are a competent vector for JCV while the virus failed to infect *An. quadrimaculatus* [28]. In contrast, our results showed relatively high infectivity, yet also low dissemination and no transmission for representative JCV strains from both lineages. Together, these results suggest that despite the association with JCV in nature, *An. quadrimaculatus* mosquitoes appear to play little role in horizontal transmission of the virus. These results stand in contrast to the orthobunyavirus CVV, for which *An. quadrimaculatus* are highly competent and likely to play a primary role in transmission, particularly for the emergent lineage 2 CVV [22]. However, it has been demonstrated that forced salivation methods tend to underestimate virus transmission, so low-level transmission in natural systems remains plausible [29]. Moreover, transmission of arboviruses is influenced by interactions among viral genotype, vector genotype and environment [30, 31]. Since we used a colony of unknown generations of *An. quadrimaculatus* under controlled environmental conditions, further variability in competence for JCV could exist with field populations subjected to distinct or fluctuating environments.

*Aedes albopictus* is one of the most successful invasive species globally and continues to expand its geographic distribution both in the US and other countries [32, 33]. In NYS, *Ae. albopictus* populations are well established in Long Island, the Hudson Valley and New York City counties; however, the species is still undergoing population invasion and establishment [20, 34]. Furthermore, endemic viruses such as CVV, eastern equine encephalitis virus, JCV, West Nile virus, Potosi virus and LACV have been isolated from this species in the US [35, 36]. However, the role of *Ae. albopictus* as a vector of local arboviruses is not yet fully elucidated [36, 37]. Grimstad et al. reported JCV transmission at 14 dpi by *Ae. albopictus* [38]. Additional studies assessing vector competence of local *Aedes* mosquitoes for JCV broadly demonstrate transmissibility [28, 39, 40]. Importantly, we now demonstrate transmission of both JCV lineages by *Ae. albopictus* and high competence at a large range of doses. These data have important implications for infectiousness during viremic phases of primary hosts, but additionally suggest the potential for less competent hosts to contribute to transmission cycles. Surprisingly, we also found that JCV is consistently better transmitted at 7 dpi relative to 14 dpi. Since there is no evidence that JCV or other arboviral infections are regularly cleared from mosquitoes, these data suggest that virulence of JCV in vectors could impact longevity and transmissibility.

Although JCV cases are more frequently diagnosed in the upper Midwest, prevalence in the Eastern US has been increasing [7]. In addition, in North Carolina the only documented case of JCV occurred in a liver transplant patient [41]. Furthermore, JCV has consistently been detected from diverse mosquito species in the Northeast, with *Aedes* species most strongly associated with the virus [10, 17, 19]. Three exotic invasive mosquito species (*Ae. albopictus*, *Ae. aegypti* and *Ae japonicus*) have been shown to be competent vectors for local endemic orthobunyaviruses including JCV, LACV and CVV [21, 42–45]. Together, these data suggest that the transmission risk of these native mosquito-borne diseases may be increased by the introduction and establishment of invasive mosquito species.

## Conclusion

To the best of our knowledge, our study provides the first evidence of infection of JCV in *An. quadrimaculatus* and vector competence of *Ae. albopictus* for both lineages. Establishment of *Ae. albopictus* in the Northeast could increase the threat of JCV transmission in the region.

## Data Availability

Data generated in this study are available from the corresponding authors upon reasonable request.

## Abbreviations

dpi: Days post-infection
Ae: *Aedes*
An: *Anopheles*
JCV: Jamestown Canyon virus
LACV: La Crosse virus
CVV: Cache Valley virus
N: Number
IR: Infection rate
DR: Dissemination rate
TR: Transmission rates
US: United States
NYS: New York State
